# Self-Averaging Property of Minimal Investment Risk of Mean-Variance Model

**DOI:** 10.1371/journal.pone.0133846

**Published:** 2015-07-30

**Authors:** Takashi Shinzato

**Affiliations:** Mori Arinori Center for Higher Education and Global Mobility, Hitotsubashi University, Kunitachi, Tokyo, Japan; Universidad Veracruzana, MEXICO

## Abstract

In portfolio optimization problems, the minimum expected investment risk is not always smaller than the expected minimal investment risk. That is, using a well-known approach from operations research, it is possible to derive a strategy that minimizes the expected investment risk, but this strategy does not always result in the best rate of return on assets. Prior to making investment decisions, it is important to an investor to know the potential minimal investment risk (or the expected minimal investment risk) and to determine the strategy that will maximize the return on assets. We use the self-averaging property to analyze the potential minimal investment risk and the concentrated investment level for the strategy that gives the best rate of return. We compare the results from our method with the results obtained by the operations research approach and with those obtained by a numerical simulation using the optimal portfolio. The results of our method and the numerical simulation are in agreement, but they differ from that of the operations research approach.

## Introduction

Investment is one of the most common economic activities, and it is defined as an activity in which it is expected that future remuneration will more than repay the cost [1–3]. The uncertainty involved in investments cannot be removed, and in general, a greater risk corresponds to a greater expected return. In this paper, we consider the portfolio optimization problem, which is a mathematical formulation of risk management for investments. The portfolio optimization problem is based on the framework of risk diversification management, which was introduced by Markowitz in 1952; it is the topic of some of the most important and most active research in mathematical finance, and various models have been proposed [2–5]. For instance, Markowitz proposed a rule for investing in several securities in order to diversify. For example, this rule states that when the expected return and the invested assets are constant, the best strategy minimizes the variance of the return (investment risk). Markowitz also analytically derived the investment strategy which minimizes investment risk. Konno and Yamazaki proposed the mean-absolute deviation model, whose risk function is not defined by the variance of the return but as the sum of the absolute error in each period; it has also been shown that the optimal solutions of the mean-variance model and that of the mean-absolute deviation model are in agreement [4]. Rockafellar and Uryasev proposed an expected shortfall model that is based on an index that measures the risk of not being less than a chosen confidence level; this model considers the downside risk of stochastically fluctuating gross earnings [5].

In recent decades, the portfolio optimization problem has been studied using analytical approaches that were developed in cross-disciplinary fields other than operations research [6–9]. Ciliberti and Mézard used the replica analysis method developed in spin glass theory to analyze the typical behaviors of the risk functions of the mean-absolute deviation model and the expected shortfall model [6]. Pafka and Kondor compared the distribution of the eigenvalues of the variance-covariance matrix defined by the return rate obtained from the dealings market with the limit distribution acquired by assuming an independent return rate; they also quantitatively analyzed the correlation between assets using random matrix theory, which was developed in mathematical statistics and quantum chaos [7]. Shinzato and Yasuda used a belief propagation method that was developed as a decoding algorithm to create an algorithm which can derive the optimal solution, with computational complexity that is proportional to the square of the number of investment assets [8]. Wakai, Shinzato, and Shimazaki analyzed the typical behaviour when using minimal investment risk and the concentrated investment level of Markowitz’s mean-variance model for the cases in which the return rates of the random matrix ensemble were independently and identically distributed from a normal distribution, a uniform distribution, and an exponential distribution [9].

Although studies have used methods from random matrix theory and statistical mechanical informatics to analyze the potential risks for the portfolio optimization problem [10], this has been done without a mathematical proof that the self-averaging property of investment risk and the concentrated investment level can be used to effectively evaluate the optimal solution (self-averaging will be further discussed below) [11]. However, it is not obvious that these indicators are self-averaging. Furthermore, if they are self-averaging, the potential risk of an investment system can be analyzed in this way, but it is important to determine whether this is so since the results are not always in agreement with those produced using operations research. Thus it is necessary to consider these problems systematically.

Therefore, in this paper, we provide a mathematical proof of the self-averaging property and discuss the validity of the analytical procedure that is widely used in operations research approaches to the portfolio optimization problem. To do this, we reformulate the portfolio optimization problem using a probabilistic framework. We also consider two scenarios, neither of which have been previously addressed, for the optimization of stochastic phenomena. We introduce the concept of self-averaging, and we then use it to analyze the potential risk of an investment system and determine the optimal investment strategy. We validate our proposed approach by comparing it with the results obtained by the standard operations research method and with those of a numerical simulation, and finally, we summarize the problems of using the operations research approach for a problem with this mathematical structure.

This paper is organized as follows. In the next section, we mathematically formulate the portfolio optimization problem and discuss an easy game that optimizes stochastic phenomena; this presents the viewpoint that we will use to analyze the potential risk of an investment system. The following section presents the concepts we will use, such as those from statistical mechanics and probabilistic inequalities, and summarizes the self-averaging property, which is an important feature of the optimal investment strategy. We present our results and compare them with those of other methods, as discussed above. In the final section, we present a summary and discuss areas of future work.

## Model setting and optimization for stochastic phenomena

### Markowitz’s mean-variance portfolio selection

In this subsection, we present the mean-variance model, which is one of the most commonly used models for the portfolio optimization problem. We begin by considering a stable investment market with *N* investment outlets, where *w*_*k*_ represents the portfolio (or investment ratio) of asset *k*(= 1, ⋯, *N*), and $x_{k\mu }^{\prime }$ denotes the return rate of asset *k* in scenario *μ*(= 1, ⋯, *p*). However, for simplicity, we do not include short sales, that is, −∞ < *w*_*k*_ < ∞, and we assume that the probability distribution of the return rate is known for each asset. In Markowitz’s mean-variance model, given *p* scenarios, the investment risk is defined to be the sum of squares of the difference between the gross return for a scenario $\sum_{k=1}^{N}x_{k\mu }^{\prime }w_{k}$ and its expectation $\sum_{k=1}^{N}E[x_{k\mu }^{\prime }]w_{k}$; determining an investment strategy by minimizing the risk creates a hedge. That is to say, the investment risk of a portfolio with *N* assets **w** = (*w*_1_, ⋯, *w*_*N*_)^T^ ∈ **R** ^*N*^ is defined as

$$\begin{array}{ccc} 𝓗(w|X) & = & \frac{1}{2N}\sum_{\mu =1}^{p}{\left(\sum_{k=1}^{N}x_{k\mu }^{\prime }w_{k}-\sum_{k=1}^{N}E\left[x_{k\mu }^{\prime }\right]w_{k}\right)}^{2}\\ & = & \frac{1}{2}\sum_{\mu =1}^{p}{\left(\frac{1}{\sqrt{N}}\sum_{k=1}^{N}x_{k\mu }w_{k}\right)}^{2}, \end{array}$$ (1)

where T denotes the transpose of a matrix or vector, and *E*[*f*(*x*)] is the expectation of *f*(*x*). Since we have assumed that the probability distribution of the return rate of each asset is known, we represent the return rate as $x_{k\mu }=x_{k\mu }^{\prime }-E[x_{k\mu }^{\prime }]$ and the return rate matrix as $X=\left\{\frac{x_{k\mu }}{\sqrt{N}}\right\}\in {𝓜}_{N\times p}$. Also, note that although we introduce the coefficient $\frac{1}{2N}$ in Eq (1) for simplicity of the discussion below, since $\sum_{k=1}^{N}x_{k\mu }w_{k}$ is the summation of *N* random variables *x*_*kμ*_ *w*_*k*_ (*w*_*k*_ can be interpreted as the coefficient of a random variable *x*_*kμ*_), even if we do not assume that the return rates of the assets are independent, if **w** is fixed, the correlation between the returns is small, and the third and higher moments of the return rate are finite, then we expect that as the number of investment outlets *N* increases, $v_{\mu }=\frac{1}{\sqrt{N}}\sum_{k=1}^{N}x_{k\mu }w_{k}$ asymptotically approaches a multidimensional Gaussian distribution according to the central limit theorem.

In the mean-variance model, in the absence of constraints (such as budgets), an obvious optimal portfolio is obtained by minimizing the risk function 𝓗(**w**∣*X*) with *w*_1_ = ⋯ = *w*_*N*_ = 0. Since this is equivalent to not investing, there is no investment risk; however, in this paper, we use the budget constraint

$$\begin{array}{ccc} \sum_{k=1}^{N}w_{k} & = & N. \end{array}$$ (2)

Moreover, although in the actual management of assets, it is necessary to impose expected return restrictions in addition to budget constraints, for simplicity, we will consider only budget constraints. Therefore, the portfolio optimization problem is formulated as determining the portfolio **w** that minimizes 𝓗(**w**∣*X*), that is, the risk function in Eq (1) with the constraint of Eq (2). In the case of *p* > *N*, the optimal solution can be analytically determined:

$$\begin{array}{ccc} w & = & \frac{NJ^{-1}e}{e^{T}J^{-1}e}, \end{array}$$ (3)

where the unit vector **e** = (1, 1, ⋯, 1)^T^ ∈ **R** ^*N*^ and *J* ^−1^ is the inverse of the variance-covariance matrix *J* = {*J*_*ij*_} = *XX* ^T^ ∈ 𝓜_*N* × *N*_, where element *i*, *j* of matrix *J* is

$$\begin{array}{ccc} J_{ij} & = & \frac{1}{N}\sum_{\mu =1}^{p}x_{i\mu }x_{j\mu }. \end{array}$$ (4)

If *p* ≤ *N*, then since matrix *J* is not a regular matrix, the optimal solution of this portfolio optimization problem cannot be uniquely determined.

Using the definition of 𝓗(**w**∣*X*) in Eq (1), for each scenario *μ*, we can estimate the sum of squares of the difference between the gross earnings $\sum_{k=1}^{N}x_{k\mu }^{\prime }w_{k}$ and its expectation $\sum_{k=1}^{N}E[x_{k\mu }^{\prime }]w_{k}$; this can be interpreted as the investment potential of portfolio **w**, and the concentrated investment level *q*_*w*_ is defined as follows [6, 8, 9]:

$$\begin{array}{ccc} q_{w} & = & \frac{1}{N}\sum_{k=1}^{N}w_{k}^{2}. \end{array}$$ (5)

With an equipartition investment strategy **w** = (1, 1, ⋯, 1)^T^ ∈ **R** ^*N*^, we obtain *q*_*w*_ = 1; with a concentrated investment strategy, for example, investing only in asset 1, **w** = (*N*, 0, ⋯, 0)^T^ ∈ **R** ^*N*^, so *q*_*w*_ = *N* is obtained; if one investor invests equally in *m* of *N* possible outlets, *q*_*w*_ = *N*/*m*. Thus we have

$$\begin{array}{ccc} q_{w}-1 & = & \frac{1}{N}\sum_{k=1}^{N}w_{k}^{2}-{\left(\frac{1}{N}\sum_{k=1}^{N}w_{k}\right)}^{2}\\ & = & \frac{1}{N}\sum_{k=1}^{N}{\left(w_{k}-\frac{1}{N}\sum_{k^{\prime }=1}^{N}w_{k^{\prime }}\right)}^{2}, \end{array}$$ (6)

and as portfolio **w** approaches equipartition, *q*_*w*_ decreases to 1, and as it approaches a concentrated investment strategy, *q*_*w*_ increases.

We note that although $\sum_{k=1}^{N}w_{k}=1$ is widely used as a budget constraint in operations research, we do not use one in this paper. Since the optimal solution to the portfolio optimization problem with a budget constraint that is widely used in operations research is ${\text{w}}^{1}={\left(w_{1}^{1},w_{2}^{1},\cdots ,w_{N}^{1}\right)}^{\text{T}}\in {\text{R}}^{N}$, and the optimal solution defined in Eq (2) is ${\text{w}}^{N}={\left(w_{1}^{N},w_{2}^{N},\cdots ,w_{N}^{N}\right)}^{\text{T}}\in {\text{R}}^{N}$, the relation $w_{i}^{1}/w_{j}^{1}=w_{i}^{N}/w_{j}^{N}$ is proved, that is, in the optimal portfolios of each method, the investment ratios are in agreement. Furthermore, the concentrated investment level *q*_*w*_ can be interpreted as an indicator of diversification when using the budget constraint of Eq (2).

### Optimization for Stochastic Phenomena

In this subsection, we consider this optimization problem from a different viewpoint. We analyze the behaviour of minimal investment risk *ɛ* and the concentrated investment level *q*_*w*_ of the mean-variance model, and we discuss the optimization of stochastic phenomena which have not been addressed in the operations research approach to this problem. Let us consider the following variant of the well-known game of rock-paper-scissors. Rule 1: Two subjects, Alice and Bob, play rock-paper-scissors 300 times. Rule 2: Alice can freely choose to display rock, paper, or scissors. On the other hand, Bob’s choice is randomly assigned by the toss of a fair dice: rock when the dice shows 1 or 2, paper for 3 or 4, and scissors for 5 or 6. Moreover, Alice knows that Bob’s choice is randomly and independently determined by the dice. Rule 3: The winner adds a point, the loser subtracts a point, and if they tie, there is no change to the score. We now consider whether it is expected that Alice will win overall.

#### (a) Two subjects simultaneously hold out their hands to indicate rock, paper, or scissors

First, we consider the ordinary case. Since Alice does not know Bob’s choice, she assumes that each possibility has equal probability. Thus, if Alice also chooses according to a roll of a dice, the expected total acquired score would be 0. Similarly, if Alice chooses only rock, the expected score would be 0. We will use the following notation: *r*_*A*_ (resp. *r*_*B*_) is the probability Alice (resp. Bob) chooses rock, *p*_*A*_ (resp. *p*_*B*_) is the probability Alice (resp. Bob) chooses paper, and *s*_*A*_ (resp. *s*_*B*_) is the probability Alice (resp. Bob) chooses scissors; note that for each player, the expectation of the total acquired score is 0. That is, if Alice does not have prior knowledge of Bob’s choice, neither of them can win (for a sufficient number of trials), and the expectation is that they tie. However, if the probabilities of Bob’s choice are not equal (e.g., (*r*_*B*_, *p*_*B*_, *s*_*B*_) = (2/3,1/6,1/6)), then Alice should choose (*r*_*A*_, *p*_*A*_, *s*_*A*_) = (0,1,0). In this case, the expected total acquired score for Alice is 150. Generally speaking, even if Alice does not have prior knowledge of Bob’s choice, if she knows the probabilities of his choices, she can choose in such a way that maximizes her expected score.

#### (b) Alice has prior knowledge of Bob’s choice

We now consider the case where Alice makes her choice after learning what Bob will display. Her goal is to maximize the expectation of her total score. With added constraints, her expected total score will be larger than 0; without constraints, it will be 300.

#### (c) There is a constraint on the number of times that rock, paper, and scissors can each be chosen

We now consider the case where Alice’s choices are constrained; for instance, they must each be chosen an equal number of times (i.e., 100 times). With this constraint, the expected total acquired score is 0 for case (a) (Alice does not have prior knowledge of Bob’s choice), but for case (b) (Alice has prior knowledge of Bob’s choice), it is 500/3. That is, if she has prior knowledge, she can take protective action.

#### (d) Five sets of 300 sessions

Finally, we consider the case that two subjects play five sets of 300 games. If Alice has no prior knowledge of Bob’s choice, her expected total acquired score is again 0. If she has prior knowledge and there are no constraints, her expected score is 1500. If there is a constraint such that Alice must make the same choice each time, her expected score is 0 for case (a) (Alice does not have prior knowledge of Bob’s choice), but 5000/9 for case (b) (Alice has prior knowledge of Bob’s choice). In case (c) (Alice’s choices are constrained), it is easy to see that the expectation of Alice’s total score for case (a) is not larger than it is for case (b).

In conclusion, for both case (c) (Alice’s choices are constrained) and case (d) (five sets of 300 games and Alice makes the same choice each time), if Alice has prior knowledge of Bob’s choice, her score will be higher than if she has no such knowledge. That is, if Alice has prior knowledge, she can produce a better strategy.

We would like to make one more point, which will be further discussed below. Cases (a) and (b) (respectively, Alice does not or does have prior knowledge of Bob’s choice) are similar to the discussion of annealed and quenched disorder systems in statistical mechanics [10, 12]. In an annealed disorder system, the indicator *f*(**w**∣*X*) is first averaged using a random *X* in the disordered system, and then the averaged indicator *E*[*f*(**w**∣*X*)] is optimized in order to assess the behaviour of the system. In the rock-paper-scissors example, the indicator *f*(**w**∣*X*) corresponds to the total acquired score of Alice, **w** corresponds to Alice’s choices (or strategy), the random *X* corresponds to Bob’s choices, and case (a) corresponds to the annealed disorder system. On the other hand, in a quenched disorder system, *f*(**w**∣*X*) is first optimized subject to a restriction and a random *X* which is included in the disordered system, and then the optimized indicator is averaged using the random *X* in order to assess the behaviour of this system; this corresponds to case (b) of the rock-paper-scissors example. More generally, when optimizing an indicator *f*(**w**∣*X*) for a stochastic phenomena, it matters in which order the averaging and optimizing occur. When maximizing, it is necessary to precisely estimate two kinds of indicators, *f*_*a*_ = max_**w**_ *E*[*f*(**w**∣*X*)] and *f*_*q*_ = *E*[max_**w**_ *f*(**w**∣*X*)]. Since for any **w**, max_**w**_ *f*(**w**∣*X*) ≥ *f*(**w**∣*X*) holds for any random *X*, to find the relative magnitude of *f*_*a*_ and *f*_*q*_, one averages both sides and then maximizes the right-hand side. The left-hand side does not need to be maximized because a definite value is obtained for *f*_*q*_ ≥ *f*_*a*_. When minimizing, we have *f*_*a*_ = min_**w**_ *E*[*f*(**w**∣*X*)] and *f*_*q*_ = *E*[min_**w**_ *f*(**w**∣*X*)], and so in way similar to the above, we obtain *f*_*a*_ ≥ *f*_*q*_, that is,

$$\begin{array}{c} \min_{w}E\left[f\left(w|X\right)\right]\geq E[\min_{w}f\left(w|X\right)]. \end{array}$$ (7)

### Operations research approach for portfolio optimization

From the above argument, we see that optimization of stochastic phenomena is handled differently for an annealed disorder system than it is for a quenched disorder system. Using this core concept, let us reconsider the portfolio optimization problem. In the standard analytical approach of operations research to the portfolio optimization problem, one first averages the risk function 𝓗(**w**∣*X*) with the return rate on the assets and then minimizes the expectation of the risk function *E*[𝓗(**w**∣*X*)] with a budget constant. Here, for simplicity, we presume that return rate is independently and identically distributed with a standard normal distribution. Thus, the expectation of the correlation between asset *i* and asset *j* is

$$\begin{array}{ccc} E[J_{ij}] & = & \left\{ \begin{array}{cc} \frac{p}{N} & i=j\\ 0 & i\neq j. \end{array}\right. \end{array}$$ (8)

Using this, the expected investment risk function *E*[𝓗(**w**∣*X*)] with a return rate on the assets is

$$\begin{array}{ccc} E[𝓗(w|X)] & = & \frac{\alpha }{2}\sum_{k=1}^{N}w_{k}^{2}, \end{array}$$ (9)

where the ratio *α* = *p*/*N* is used. In addition, from the symmetry of this model, the optimal investment strategy of Eq (9), using the budget constraint of Eq (2), describes an equipartition investment strategy. The minimum expected investment risk per asset ${ɛ}^{\text{OR}}=\frac{1}{N}{\text{min}}_{\text{w}}E[𝓗(\text{w}|X)]$ is evaluated as follows:

$$\begin{array}{ccc} \varepsilon^{\text{OR}} & = & \frac{\alpha }{2}. \end{array}$$ (10)

The concentrated investment level $q_{w}^{\text{OR}}$ is

$$\begin{array}{ccc} q_{w}^{\text{OR}} & = & 1. \end{array}$$ (11)

This analytical approach, which is widely used in operations research, does not provide insight into the optimal investment strategy in an actual market; the reason is not just that the model was simplified by assuming the return rate is independently and identically distributed with a standard normal distribution. Since this analytical approach is equivalent to case (a) in the rock-paper-scissors game with two subjects and the annealed disorder system, it is not clear that this approach could be used to minimize the investment risk 𝓗(**w**∣*X*) with respect to a realistic individual return rate matrix *X*, that is, **w** = argmin_**w**_𝓗(**w**∣*X*). In particular, the equality argmin_**w**_𝓗(**w**∣*X*) = argmin_**w**_ *E*[𝓗(**w**∣*X*)] is not always satisfied. As we discussed with the rock-paper-scissors game, if we average the investment risk with the return rate, we can avoid the complication of optimizing individual return rates; on the other hand, this approach does not evaluate the optimal strategy based on individual return rates. Even though it is not guaranteed mathematically that the solution to the minimal expected investment risk optimizes the investment risk for each set of return rates 𝓗(**w**∣*X*), this approach is widely used in operations research and might provide a misleading investment strategy.

On the other hand, let us consider case (b) in the rock-paper-scissors game with two subjects and the quenched disorder system. In a stable investment market, even if at *μ* = 0 one had prior information about the probability distribution of the return rate of each asset during the next period (*μ* = 1 to *μ* = *p*), since it is not possible to know the actual return rate, it is difficult to select the optimal investment strategy. However, if we have prior information about the return rates, as discussed in the rock-paper-scissors example, we can minimize the investment risk and obtain an optimal investment strategy. In particular, if *p*/*N* ≤ 1, since it is well known that the optimal solution is a linear sum of the eigenvectors of the minimal eigenvalue of the variance-covariance matrix *J*, the investment risk per asset is 0, since the minimal eigenvalue of the matrix *J* is 0 since *J* is nonsingular. We next consider the concentrated investment level *q*_*w*_. Let 𝓥 be variance of the sample variance $\frac{1}{p}\sum_{\mu =1}^{p}x_{k\mu }^{2}$; it is evaluated as follows:

$$\begin{array}{ccc} 𝓥 & = & E[{\left(\frac{1}{p}\sum_{\mu =1}^{p}x_{k\mu }^{2}-\frac{1}{p}\sum_{\mu =1}^{p}E\left[x_{k\mu }^{2}\right]\right)}^{2}]\\ & = & \frac{2}{N\alpha }. \end{array}$$ (12)

When *α* = *p*/*N* is small, 𝓥 is large, and one should invest heavily in blue-chip assets for which the return rates have smaller sample variances than those of the other *N* investment outlets; in this way, the risk is decreased, and the optimal investment strategy is asymptotically close to a concentrated investment strategy, namely *q*_*w*_ ≫ 1.

When *p*/*N* > 1, using the optimal solution for Eq (3), the two indicators can be analytically assessed. That is, the minimal investment risk per asset *ɛ*(*X*) and the concentrated investment level *q*_*w*_(*X*) can be written as follows:

$$\begin{array}{ccc} \varepsilon (X) & = & \frac{1}{N}𝓗(\frac{NJ^{-1}e}{e^{T}J^{-1}e}|X)\\ & = & \frac{N}{2(e^{T}J^{-1}e)}, \end{array}$$ (13)

$$\begin{array}{ccc} q_{w}\left(X\right) & = & \frac{1}{N}{\left(\frac{NJ^{-1}e}{e^{T}J^{-1}e}\right)}^{T}(\frac{NJ^{-1}e}{e^{T}J^{-1}e})\\ & = & \frac{Ne^{T}J^{-2}e}{{\left(e^{T}J^{-1}e\right)}^{2}}, \end{array}$$ (14)

where we use the explicit return rate matrix $X=\left\{\frac{x_{k\mu }}{\sqrt{N}}\right\}\in {𝓜}_{N\times p}$ as the argument since these indicators depend on the return rate matrix. The variance-covariance matrix *J* = *XX* ^T^ ∈ 𝓜_*N* × *N*_ has already been defined. In actual investments, assuming fair dealing, since we do not have prior knowledge of the actual return rate, we cannot precisely determine the two indicators. However, we can evaluate the previous risk in an investment system and thus support the strategy of an investor. In order to provide useful insight, we need to precisely analyze *ɛ*(*X*) and *q*_*w*_(*X*). For the reasons noted here, we assume that during the initial period, we have prior knowledge of the return rate; although this assumption is impossible, we note that we will show below that this assumption is not required to evaluate the potential of an investment system. Although we need to assess the optimal solution or the inverse of the variance-covariance matrix in order to assess the potential risk of an investment market, it is difficult to do this since the computational complexity of finding the inverse matrix is proportional to the cube of the matrix size *N*, which is the number of investment outlets. In addition, we need to find each inverse matrix for each return rate in order to evaluate the minimal investment risk of each set; however, if the minimal investment risk randomly fluctuates with the return rates, we would need to average the minimal investment risk with the return rate set *X*. We now note that we can use the self-averaging property to simplify evaluation of the potential investment risk.

## Preliminaries

We first prepare some mathematical tools to enable discussion of the self-averaging property of the minimal investment risk.

### Statistical mechanics

First, using the Boltzmann distribution of the inverse temperature *β*(> 0), which is widely used in statistical mechanics [13], the posterior probability of portfolio **w** given return rate matrix *X*, *P*(**w**∣*X*), is defined as follows:

$$\begin{array}{ccc} P(w|X) & = & \frac{P_{0}\left(w\right)e^{-\beta 𝓗(w|X)}}{Z(\beta ,X)}, \end{array}$$ (15)

where the prior probability *P*_0_(**w**) is 1 if portfolio **w** satisfies Eq (2), and it is 0 otherwise; *e* ^−*β*𝓗(**w**∣*X*)^ is the likelihood function; and *Z*(*β*, *X*), the partition function, is a normalized constant and is defined as follows:

$$\begin{array}{ccc} Z(\beta ,X) & = & \int_{-\infty }^{\infty }dwP_{0}\left(w\right)e^{-\beta 𝓗(w|X)}. \end{array}$$ (16)

From this, it is found that the posterior probability *P*(**w**∣*X*) satisfies the property of a probability measure, that is, *P*(**w**∣*X*) ≥ 0 and $\int_{-\infty }^{\infty }d\text{w}P(\text{w}|X)=1$. Furthermore, it is well known that w* = arg max_**w**_ *P*(**w**∣*X*), which is obtained using the maximum a posteriori estimation, is consistent with the portfolio obtained by minimizing the investment risk function 𝓗(**w**∣*X*). By taking the limit of the inverse temperature *β*, we obtain

$$\begin{array}{ccc} \lim_{\beta \to \infty }P\left(w|X\right) & = & \prod_{i=1}^{N}\delta \left(w_{i}-w_{i}^{*}\right), \end{array}$$ (17)

where $w_{i}^{*}$ is the optimal investment ratio for asset *i*. Thus, we can average the portfolio **w** and the investment risk 𝓗(**w**∣*X*) using the a posteriori probability *P*(**w**∣*X*) and allow the inverse temperature *β* to become sufficiently large:

$$\begin{array}{ccc} w^{*} & = & \lim_{\beta \to \infty }\int_{-\infty }^{\infty }dwP\left(w|X\right)w \end{array}$$ (18)

$$\begin{array}{ccc} 𝓗(w^{*}|X) & = & \lim_{\beta \to \infty }\int_{-\infty }^{\infty }dwP\left(w|X\right)𝓗\left(w|X\right), \end{array}$$ (19)

where *δ*(*u*) is the Dirac delta function and this holds for any function *f*(*x*) such that $f(x)=\int_{-\infty }^{\infty }dyf(y)\delta (y-x)$ (see appendix 2). From this reformulation and using the posterior probability defined in Eq (15), the portfolio optimization problem can be solved using the framework of probabilistic reasoning.

### Chernoff inequality

Next, we introduce one of the probability inequalities, the Chernoff inequality, as follows. For a random variable *Y* with known probability measure and a constant number *η*, the probability that *η* ≤ *Y* satisfies the following inequality for any *u* > 0 is [14, 15]:

$$\begin{array}{ccc} Pr[\eta \leq Y] & \leq & e^{-u\eta }E\left[e^{uY}\right]. \end{array}$$ (20)

This can be easily proved; for example, consider the step function Θ(*W*), which is 1 if *W* ≥ 0 and 0 otherwise. First, for *u* > 0, we obtain Θ(*W*) ≤ *e* ^*uW*^. From this, we derive *Pr*[*η* ≤ *Y*] = *E*[Θ(*Y* − *η*)] ≤ *e* ^−*uη*^ *E*[*e* ^*uY*^]. In addition, for *Pr*[*η* ≥ *Y*], we obtain *Pr*[*η* ≥ *Y*] ≤ *e* ^−*uη*^ *E*[*e* ^*uY*^] for *u* < 0.

From Eq (20), we could derive a tighter upper bound. Since the right-hand side in Eq (20) is guaranteed for an arbitrary *u* > 0, there necessarily exists a minimum value for the right-hand side for any *u* > 0, and we obtain

$$\begin{array}{ccc} Pr[\eta \leq Y] & \leq & \min_{u>0}\{e^{-u\eta }E\left[e^{uY}\right]\}\\ & = & e^{-R(\eta )}. \end{array}$$ (21)

Here *R*(*η*) is the rate function and is defined as

$$\begin{array}{ccc} R(\eta ) & = & \max_{u>0}\{u\eta -\log E[e^{uY}]\}. \end{array}$$ (22)

The cumulative generating function *ϕ*(*u*) = log*E*[*e* ^*uY*^] is a convex function of *u*, and the rate function *R*(*η*), defined by the Legendre transformation of a convex function, is also a convex function. It is also known that *R*(*η*) is nonnegative, *R*(*η*) = 0 if *η* ≤ *E*[*Y*], and *R*(*η*) > 0 if *η* > *E*[*Y*]. These properties of the rate function are proved in appendix 1.

### Self-averaging property supported by large deviation theory

When the portfolio **w** depends on the posterior probability *P*(**w**∣*X*) defined in Eq (15), the probability that the investment risk per asset $\frac{1}{N}𝓗(\text{w}|X)$ is less than or equal to a constant number $\tilde{ɛ}$ satisfies the Chernoff inequality; that is, $Pr\left[\frac{1}{N}𝓗(\text{w}|X)\leq \tilde{ɛ}\right]=E\left[\Theta \left(N\tilde{ɛ}-𝓗(\text{w}|X)\right)\right]=\int_{-\infty }^{\infty }d\text{w}P(\text{w}|X)\Theta \left(N\tilde{ɛ}-𝓗(\text{w}|X)\right)$ satisfies

$$\begin{array}{ccc} Pr[\frac{1}{N}𝓗\left(w|X\right)\leq \tilde{\varepsilon }] & \leq & E[e^{N\tilde{\beta }\tilde{\varepsilon }-\tilde{\beta }𝓗\left(w|X\right)}]\\ & = & e^{N\tilde{\beta }\tilde{\varepsilon }}\frac{Z(\beta +\tilde{\beta },X)}{Z(\beta ,X)}. \end{array}$$ (23)

Here, $\tilde{\beta }$ is a positive number and $Z(\beta +\tilde{\beta },X)$ is defined by Eq (16) (with *β* replaced by $\beta +\tilde{\beta }$). Thus, the probability inequality of the tighter upper bound of Eq (23) is derived using the following rate function:

$$\begin{array}{ccc} R_{+}\left(\beta ,\tilde{\varepsilon },X\right) & = & \max_{\tilde{\beta }>0}\{-\tilde{\beta }\tilde{\varepsilon }-\frac{1}{N}\log Z\left(\beta +\tilde{\beta },X\right)+\frac{1}{N}\log Z\left(\beta ,X\right)\}, \end{array}$$ (24)

and we have $Pr\left[\frac{1}{N}𝓗(\text{w}|X)\leq \tilde{ɛ}\right]\leq e^{-NR_{+}(\beta ,\tilde{ɛ},X)}$ [16]. In a similar way, for the probability of $\frac{1}{N}𝓗(\text{w}|X)\geq \tilde{ɛ}$, $Pr\left[\frac{1}{N}𝓗(\text{w}|X)\geq \tilde{ɛ}\right]$, thus $Pr\left[\frac{1}{N}𝓗(\text{w}|X)\geq \tilde{ɛ}\right]\leq e^{-NR_{-}(\beta ,\tilde{ɛ},X)}$ is also obtained, where we use the rate function

$$\begin{array}{ccc} R_{-}\left(\beta ,\tilde{\varepsilon },X\right) & = & \max_{\tilde{\beta }<0}\{-\tilde{\beta }\tilde{\varepsilon }-\frac{1}{N}\log Z\left(\beta +\tilde{\beta },X\right)+\frac{1}{N}\log Z\left(\beta ,X\right)\}. \end{array}$$ (25)

In order to analyze the rate functions in Eqs (24) and (25), it is also necessary to assess $\frac{1}{N}\text{log}Z(\beta +\tilde{\beta },X)$ and $\frac{1}{N}\text{log}Z(\beta ,X)$, which depend on the return rate matrix *X*. Based on the definition in Eq (16), assessing these partition functions analytically is more difficult than assessing the optimal solution analytically. In order to resolve this difficulty, we consider the cumulative distribution of $\frac{1}{N}\text{log}Z(\beta ,X)$ or the Helmholtz free energy *f*(*β*, *X*), as defined in the following equation:

$$\begin{array}{ccc} f(\beta ,X) & = & -\frac{1}{N\beta }\log Z\left(\beta ,X\right). \end{array}$$ (26)

The Helmholtz free energy *f*(*β*, *X*) fluctuates randomly with the probability of the return rate matrix *X*. Thus, it is necessary to evaluate the Chernoff inequality for the Helmholtz free energy and its rate function:

$$\begin{array}{ccc} Pr[f\left(\beta ,X\right)\leq \tilde{f}] & \leq & e^{Nn\beta \tilde{f}}E\left[e^{-Nn\beta f(\beta ,X)}\right]\\ & = & e^{Nn\beta \tilde{f}}E\left[Z^{n}\left(\beta ,X\right)\right], \end{array}$$ (27)

where *n* > 0 has already been defined. In a similar way, we have

$$\begin{array}{ccc} Pr[f\left(\beta ,X\right)\geq \tilde{f}] & \leq & e^{Nn\beta \tilde{f}}E\left[Z^{n}\left(\beta ,X\right)\right], \end{array}$$ (28)

where *n* < 0. In conclusion, we obtain the two probability inequalities, $Pr\left[f(\beta ,X)\leq \tilde{f}\right]\leq e^{-NR_{+}(\beta ,\tilde{f})}$ and $Pr\left[f(\beta ,X)\geq \tilde{f}\right]\leq e^{-NR_{-}(\beta ,\tilde{f})},$ where

$$\begin{array}{ccc} R_{+}\left(\beta ,\tilde{f}\right) & = & \max_{n>0}\{-n\beta \tilde{f}-\frac{1}{N}\log E\left[Z^{n}\left(\beta ,X\right)\right]\}, \end{array}$$ (29)

$$\begin{array}{ccc} R_{-}\left(\beta ,\tilde{f}\right) & = & \max_{n<0}\{-n\beta \tilde{f}-\frac{1}{N}\log E\left[Z^{n}\left(\beta ,X\right)\right]\}. \end{array}$$ (30)

In both inequalities, it is necessary to analyze $\frac{1}{N}\text{log}E[Z^{n}(\beta ,X)]$.

## Replica analysis and numerical simulation

### Similarity to the Hopfield model

In this subsection, in order to determine whether we can use replica analysis to evaluate *E*[*Z* ^*n*^(*β*, *X*)], let us consider briefly the problem of recalling a pattern stored in a neural network constructed of *N* neurons; the mathematical structure of this problem is similar to the portfolio optimization problem [10, 17]. Let *S*_*k*_ be the state of neuron *k*; then *S*_*k*_ = 1 if neuron *k* has been fired and *S*_*k*_ = −1 otherwise. Additionally, *x*_*kμ*_, (*k* = 1, ⋯, *N*, *μ* = 1, ⋯, *p*) is the memory of neuron *k* for pattern *μ* included in *p* stored patterns, and it is randomly assigned ±1 with equal probability.

Then, for *p* patterns, the Hebb rule is defined as follows:

$$\begin{array}{ccc} J_{ij} & = & \frac{1}{N}\sum_{\mu =1}^{p}x_{i\mu }x_{j\mu } \end{array}$$ (31)

where *J*_*ij*_ is the correlation between neuron *i* and neuron *j*. Thus, it is well known that the neuron state **S** that minimizes the Hamiltonian 𝓗(**S**∣*X*) in Eq (32) is consistent with each stored pattern:

$$\begin{array}{ccc} 𝓗(S|X) & = & -\frac{1}{2}S^{T}JS. \end{array}$$ (32)

If neuron state **S** is consistent with pattern 1, that is, *S*_*k*_ = *x*_*k*1_, the Hamiltonian 𝓗(**S**∣*X*) can be written as

$$\begin{array}{ccc} 𝓗(S|X) & = & -\frac{1}{2N}{\left(\sum_{k=1}^{N}x_{k1}S_{k}\right)}^{2}-\frac{1}{2N}\sum_{\mu =2}^{p}{\left(\sum_{k=1}^{N}x_{k\mu }S_{k}\right)}^{2}\\ & = & -\frac{N}{2}, \end{array}$$ (33)

where the overlap between pattern *μ* and pattern *ν* in limited by the number of neurons *N* and satisfies

$$\begin{array}{ccc} \frac{1}{N}\sum_{k=1}^{N}x_{k\mu }x_{k\nu } & = & \left\{ \begin{array}{cc} 1 & \mu =\nu \\ 0 & \mu \neq \nu \end{array}.\right. \end{array}$$ (34)

Intuitively, each pattern is orthogonal with each of the others, since the stored patterns are independent and are randomly assigned. This problem of recalling patterns stored in a neural network and of accounting for the number of identifiable patterns is called the associative memory problem, and the model defined in Eq (32) is called the Hopfield model.

In the analysis of the Hopfield model, the upper limit of the number of identifiable patterns is estimated using *E*[*Z* ^*n*^(*β*, *X*)], which evaluates the learning potential of the neural network. We also note the mathematical similarity between this model and the mean-variance model, which indicates that we could adapt the analytical approach used for the Hopfield model; that is, we could use replica analysis for the portfolio optimization problem and to assess the potential of the investment system.

### Main results obtained in replica analysis

For the detailed calculations of replica analysis, please see appendix 2. We will limit the number of investment outlets *N* such that *α* = *p*/*N* ∼ *O*(1). For *n* ∈ **N**, we have

$$\begin{array}{ccc} \Phi (n) & = & \lim_{N\to \infty }\frac{1}{N}\log E\left[Z^{n}\left(\beta ,X\right)\right]\\ & = & \underset{k,Q_{w},{\tilde{Q}}_{w}}{\mathrm{Extr}}\{-\frac{\alpha }{2}\log\det|I+\beta Q_{w}|+\frac{1}{2}\text{Tr}Q_{w}{\tilde{Q}}_{w}-\frac{1}{2}\log\det|{\tilde{Q}}_{w}|\\ & & -e^{T}k+\frac{1}{2}k^{T}{\tilde{Q}}_{w}^{-1}k,\} \end{array}$$ (35)

where $\text{k}={\left(k_{1},\cdots ,k_{n}\right)}^{\text{T}}\in {\text{R}}^{n},Q_{w}=\left\{q_{wab}\right\}\in {𝓜}_{n\times n},{\tilde{Q}}_{w}=\left\{{\tilde{q}}_{wab}\right\}\in {𝓜}_{n\times n}$, *k*_*a*_, *q*_*wab*_, and ${\tilde{q}}_{wab}$ are order parameters, **e** = (1, ⋯, 1)^T^ ∈ **R** ^*n*^ is a constant vector, *I* ∈ 𝓜_*n* × *n*_ is the identity matrix, and Extr_*A*_ *f*(*A*) are the extrema of *f*(*A*) with respect to *A*. From this, the extrema of **k**, *Q*_*w*_, and ${\tilde{Q}}_{w}$ are assessed as follows:

$$\begin{array}{ccc} k & = & {\tilde{Q}}_{w}e, \end{array}$$ (36)

$$\begin{array}{ccc} {\tilde{Q}}_{w} & = & \beta \left(\alpha -1\right)I-\frac{\beta^{2}\left(\alpha -1\right)}{1+n\beta }D, \end{array}$$ (37)

$$\begin{array}{ccc} Q_{w} & = & \frac{1}{\beta (\alpha -1)}I+\frac{\alpha }{\alpha -1}D, \end{array}$$ (38)

where *D* = **e** **e** ^T^ ∈ 𝓜_*n* × *n*_ is a square matrix, all of whose components are 1. Based on these results, we do not need to assume replica symmetry ansatz with respect to the order parameters of this model ($k_{a},q_{wab},{\tilde{q}}_{wab}$). Thus, substituting these result into Eq (35), we have

$$\begin{array}{ccc} \Phi (n) & = & -\frac{n\alpha }{2}\log\frac{\alpha }{\alpha -1}-\frac{\alpha -1}{2}\log(1+n\beta )+\frac{n}{2}-\frac{n}{2}\log\beta \left(\alpha -1\right). \end{array}$$ (39)

Here we should note that in appendix 2, we require that the replica number *n* in Eq (35) is a natural number, that is, since *E*[*Z* ^*n*^(*β*, *X*)] at replica number *n* ∈ **N** can be estimated comparatively easily, the replica number *n* in Eq (39) should also be a natural number. However, in the optimization in Eqs (29) and (30), we need to have *n* ∈ **R** to adequately discuss the solution. Thus, we assume here that the replica number *n* in Eq (39) is a real number and use this to discuss our approach in detail. In below subsection, we will compare this result with the result to justify that this is applicable.

The two rate functions are calculated as follows:

$$\begin{array}{ccc} R_{+}\left(\beta ,\tilde{f}\right) & = & \left\{ \begin{array}{cc} 0 & \frac{\alpha -1}{2}-\frac{\Lambda (\beta )}{2\beta }\leq \tilde{f}\\ \frac{\alpha -1}{2}(s-1-\log s) & \frac{\alpha -1}{2}-\frac{\Lambda (\beta )}{2\beta }>\tilde{f} \end{array}\right. \end{array}$$ (40)

$$\begin{array}{ccc} R_{-}\left(\beta ,\tilde{f}\right) & = & \left\{ \begin{array}{cc} \frac{\alpha -1}{2}(s-1-\log s) & \frac{\alpha -1}{2}-\frac{\Lambda (\beta )}{2\beta }<\tilde{f}\\ 0 & \frac{\alpha -1}{2}-\frac{\Lambda (\beta )}{2\beta }\geq \tilde{f}, \end{array}\right. \end{array}$$ (41)

where

$$\begin{array}{ccc} \Lambda (\beta ) & = & 1-\alpha \log\frac{\alpha }{\alpha -1}-\log\beta \left(\alpha -1\right) \end{array}$$ (42)

$$\begin{array}{ccc} s & = & \frac{\tilde{f}+\frac{\Lambda (\beta )}{2\beta }}{\frac{\alpha -1}{2}}. \end{array}$$ (43)

This result satisfies the properties of a rate function, as shown in appendix 1. Moreover, using Gibbs inequality, *s* − 1 − log *s* ≥ 0, if *α* > 1, in the limit as the number of investment outlets *N* becomes sufficiently large, we obtain

$$\begin{array}{ccc} Pr[f\left(\beta ,X\right)\leq \tilde{f}] & = & \left\{ \begin{array}{cc} 1 & \frac{\alpha -1}{2}-\frac{\Lambda (\beta )}{2\beta }\leq \tilde{f}\\ 0 & \frac{\alpha -1}{2}-\frac{\Lambda (\beta )}{2\beta }>\tilde{f} \end{array}\right. \end{array}$$ (44)

$$\begin{array}{ccc} Pr[f\left(\beta ,X\right)\geq \tilde{f}] & = & \left\{ \begin{array}{cc} 0 & \frac{\alpha -1}{2}-\frac{\Lambda (\beta )}{2\beta }<\tilde{f}\\ 1 & \frac{\alpha -1}{2}-\frac{\Lambda (\beta )}{2\beta }\geq \tilde{f} \end{array}.\right. \end{array}$$ (45)

From Eqs (44) and (45), since *f*(*β*, *X*) is localized around the constant $\frac{\alpha -1}{2}-\frac{\Lambda (\beta )}{2\beta }$,

$$\begin{array}{ccc} f(\beta ,X) & = & \frac{\alpha -1}{2}-\frac{\Lambda (\beta )}{2\beta } \end{array}$$ (46)

is verified for a realistic set of return rates. Namely, the Helmholtz free energy per asset *f*(*β*, *X*), which is a function of the random variable *X*, becomes a definite value in the limit of sufficiently large *N*. Thus, we have

$$\begin{array}{ccc} f(\beta ,X) & = & E[f(\beta ,X)]. \end{array}$$ (47)

In addition, because of localizing around the definite value, *f* ^*m*^(*β*, *X*) = *E*[*f* ^*m*^(*β*, *X*)] is also satisfied. This property in which a statistic or function of a random variable localizes around a definite value (or its average) is called a self-averaging property. By substituting Eq (47) into Eq (26), we obtain

$$\begin{array}{ccc} \frac{1}{N}\log Z\left(\beta ,X\right) & = & \frac{\Lambda (\beta )}{2}-\frac{\beta (\alpha -1)}{2}, \end{array}$$ (48)

and the two rate functions

$$\begin{array}{ccc} R_{+}\left(\beta ,\tilde{\varepsilon },X\right) & = & \left\{ \begin{array}{cc} +\infty & \tilde{\varepsilon }\leq \frac{\alpha -1}{2}\\ \frac{1}{2}\left(s^{\prime }-1-\log s^{\prime }\right) & \frac{\alpha -1}{2}<\tilde{\varepsilon }<\frac{\alpha -1}{2}+\frac{1}{2\beta }\\ 0 & \frac{\alpha -1}{2}+\frac{1}{2\beta }\leq \tilde{\varepsilon } \end{array}\right. \end{array}$$ (49)

$$\begin{array}{ccc} R_{-}\left(\beta ,\tilde{\varepsilon },X\right) & = & \left\{ \begin{array}{cc} \frac{1}{2}\left(s^{\prime }-1-\log s^{\prime }\right) & \frac{\alpha -1}{2}+\frac{1}{2\beta }<\tilde{\varepsilon }\\ 0 & \tilde{\varepsilon }\leq \frac{\alpha -1}{2}+\frac{1}{2\beta } \end{array},\right. \end{array}$$ (50)

where $s^{\prime }=2\beta \left(\tilde{ɛ}-\frac{\alpha -1}{2}\right)$. Thus, for a sufficiently large *N*, since the investment risk per asset $\frac{1}{N}𝓗(\text{w}|X)$ is also localized around $\frac{\alpha -1}{2}+\frac{1}{2\beta }$, the investment risk is self-averaging. Moreover, for a sufficiently large *β*, from Eq (17) we can derive the minimal investment risk, as follows:

$$\begin{array}{ccc} \varepsilon (X) & = & \frac{\alpha -1}{2}. \end{array}$$ (51)

Furthermore, since *ɛ*(*X*) is also derived analytically from an identical equation, $ɛ(X)=-{\text{lim}}_{\beta \to \infty }\frac{1}{N}\frac{\partial }{\partial \beta }\text{log}Z(\beta ,X)$, we have validated our method in another way (see appendix 2). In addition, from the self-averaging property of the investment risk, since we can ignore the dependency of the investment risk *ɛ*(*X*) on the return rate matrix *X*, we will replace *ɛ* with *ɛ*(*X*). In a similar way, *q*_*w*_(*X*) is also self-averaging, and so then

$$\begin{array}{ccc} q_{w} & = & \frac{\alpha }{\alpha -1} \end{array}$$ (52)

is obtained, where *q*_*w*_(*X*) has been replaced by *q*_*w*_.

We also note that since the minimal investment risk per asset and the concentrated investment level are both self-averaging (since their dependency on the return rate matrix *X* is ignored), we can estimate the potential of this investment system. In a stable investment market, this implies that the minimal investment risk with respect to a realistic return rate averaged over an investment period and the minimal investment risk defined by the return rate are in agreement since the minimal investment risk is self-averaging. Because of this, we do not need the assumption of the quenched disorder system that during the initial period, we have prior knowledge of the return rates that is, we only need to know a priori the previous return rates. This is another advantage of the self-averaging property.

### Comparison with results obtained by the operations research approach

In this subsection, we compare the two indicators that were derived in Eqs (10) and (11) using the analytical approach of operations research. For example, we consider ${ɛ}^{\text{OR}}=\frac{\alpha }{2}$ and $q_{w}^{\text{OR}}=1$ with the two feature indicators derived in above subsection and using the self-averaging property, that is, $ɛ=\frac{\alpha -1}{2}$ if *α* > 1 and *ɛ* = 0 otherwise, and $q_{w}=\frac{\alpha }{\alpha -1}$ if *α* > 1 and *q*_*w*_ ≫ 1 otherwise. Thus, for any *α*, we have

$$\begin{array}{ccc} \varepsilon^{\text{OR}} & \geq & \varepsilon \end{array}$$ (53)

$$\begin{array}{ccc} q_{w}^{\text{OR}} & \leq & q_{w}. \end{array}$$ (54)

First, the minimal expected investment risk *ɛ* ^OR^ is not smaller than the expected minimal investment risk *ɛ*, that is, Eq (53) is consistent with the relationship in Eq (7). Next, from both of the concentrated investment levels and using the analytical procedure of operations research, we find that the risks for each investment outlet are averaged and negated by the returns matrix. We thus conclude that the optimal strategy is equipartition investing. On the other hand, when using our proposed method, since it is possible to find the optimal solution for each investment outlet, the best return rate is found for an investment outlet that has little variation, especially if *α* is small, and this implies that the optimal strategy is concentrated investing.

Furthermore, we provide another intuitive interpretation using another mathematical argument. By the definition of *q*_*w*_ and the *N* eigenvalues of matrix *J* = *XX* ^T^ ∈ 𝓜_*N* × *N*_, *λ*_*k*_, (*k* = 1, ⋯, *N*, *λ*_1_ ≤ *λ*_2_ ≤ ⋯ ≤ *λ*_*N*_), then *q*_*w*_ = *E*[*λ* ^−2^]/(*E*[*λ* ^−1^])^2^ and where $E[\lambda^{-s}]=\frac{1}{N}\sum_{k=1}^{N}\lambda_{k}^{-s}$; see appendix 3 for the derivation. In addition, since the minimum eigenvalue of the asymptotic distribution $\lambda_{\text{min}}=1+\alpha -2\sqrt{\alpha }$ is approximately close to +0 when *α* → +1, if *m* eigenvalues are regarded as minimum eigenvalues, where *m* ∼ *O*(1), then by using L’Hôpital’s rule, we can estimate the asymptotic form of *q*_*w*_ as follows:

$$\begin{array}{ccc} q_{w} & = & \lim_{\lambda_{\min}\to +0}\frac{\frac{m}{N}\lambda_{\min}^{-2}+\frac{1}{N}\sum_{k=m+1}^{N}\lambda_{k}^{-2}}{{\left(\frac{m}{N}\lambda_{\min}^{-1}+\frac{1}{N}\sum_{k=m+1}^{N}\lambda_{k}^{-1}\right)}^{2}}\\ & = & \lim_{\lambda_{\min}\to +0}\frac{\frac{2m}{N}\lambda_{\min}^{-1}}{\frac{2m}{N}(\frac{m}{N}\lambda_{\min}^{-1}+\frac{1}{N}\sum_{k=m+1}^{N}\lambda_{k}^{-1})}\\ & = & \frac{N}{m}. \end{array}$$ (55)

Since *E*[*λ* ^−2^] increases faster than (*E*[*λ* ^−1^])^2^, *q*_*w*_ increases. This is consistent with our finding that $q_{w}=\frac{\alpha }{\alpha -1}$. Moreover, if *α* ≫ 1, then 1 ≤ *q*_*w*_ ≤ (*λ*_max_/*λ*_min_)^2^, and

$$\begin{array}{ccc} \lim_{\alpha \to \infty }{\left(\frac{1+\alpha +2\sqrt{\alpha }}{1+\alpha -2\sqrt{\alpha }}\right)}^{2} & = & 1, \end{array}$$ (56)

where the maximum asymptotic eigenvalues are $\lambda_{\text{max}}=1+\alpha +2\sqrt{\alpha }$ and $\lambda_{\text{max}}^{-s}\leq E[\lambda^{-s}]\leq \lambda_{\text{min}}^{-s}$. This is also consistent with our finding that $q_{w}=\frac{\alpha }{\alpha -1}$.

### Numerical simulation

Although we presented a theoretical discussion of the potential of an investment system, using the self-averaging property of the investment risk per asset *ɛ* and the concentrated investment level *q*_*w*_, we assumed that the replica number *n* in Eq (39) was a real number in above subsection. In the previous subsection, we presented some mathematical interpretations for our findings. However, it is not guaranteed mathematically that the replica number *n* ∈ **R** is applicable; we thus need to verify that we may use this assumption in order to legitimize the findings based on our proposed method. In this subsection, we perform a numerical simulation, and we then compare the results of our proposed method, the numerical results, and the results from the analytical operations research procedure.

In this numerical simulation, the number of investment outlets was *N* = 10^3^, and the number of scenarios was *p* ∈ [1200, 8000]; the scenario ratio was *α* ∈ [1.2,8.0]. In addition, we assessed *J* ^−1^ = (*XX* ^T^)^−1^, the inverse of the variance-covariance matrix defined by the randomly assigned return rate matrix *X*; the return rates on assets were independently and identically distributed with a standard normal distribution. We then solved Eq (3) for the optimal portfolio in order to estimate the minimal investment risk per asset *ɛ*(*X*) and the concentrated investment level *q*_*w*_(*X*). Finally, we averaged them over 100 sets of the return rate matrix.

**Fig 1:**
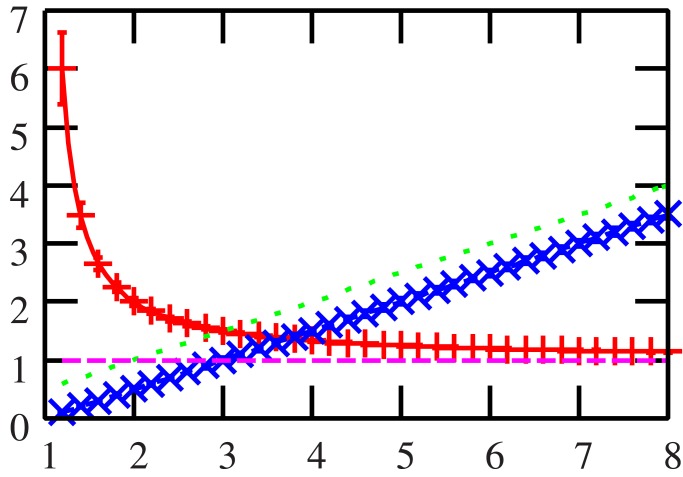
The investment risk *ɛ* and the concentrated investment level *q*_*w*_ are shown for the case in which the return rate *x*_*kμ*_ is independently and identically distributed with a standard normal distribution. The horizontal axis indicates the scenario ratio *α* = *p*/*N*, and the vertical axis shows the investment risk and the concentrated investment level *q*_*w*_. The two solid lines (results obtained by our proposed approach) and the two dotted lines (results obtained by the operations research approach) are theoretical results. The markers with error bars are the numerical results evaluated using the optimal solution according to a return rate which was randomly assigned. In the simulation, the number of investment outlets *N* was 10^3^, and we averaged 100 return rate matrices $X=\left\{\frac{x_{k\mu }}{\sqrt{N}}\right\}\in {𝓜}_{N\times p}$. This figure shows that the results obtained by our proposed approach (solid lines) and the numerical results (markers with error bars) are in agreement. On the other hand, the results obtained by the operations research approach (dotted lines) do not coincide with the others. Thus, unfortunately, the approach based on maximizing the expected utility cannot propose an optimal investment strategy.

In Fig 1, three minimal investment risks per asset and three concentrated investment levels are shown. The horizontal axis indicates the scenario ratio *α* = *p*/*N*, and the vertical axis shows the two indicators. The results of our proposed approach are indicated by solid lines, the numerical results are indicated by markers with error bars, and the results of the operations research approach are indicated by dotted lines. The results of our method (solid lines) and the numerical results (markers with error bars) are in agreement. For this numerical simulation, we considered the case in which we have a priori knowledge of the return rates. Thus, it turns out that our proposed approach can precisely assess the potential of an investment system. On the other hand, the dotted lines are based on a scenario in which the expected utility is maximized, and these results do not coincide with the others. Unfortunately, this indicates that the approach based on maximizing the expected utility is unable to determine the optimal investment strategy and may instead provide a misleading portfolio which is not guaranteed to be optimal with respect to particular set of return rates.

## Summary and future work

In this paper, we analyzed the potential of an optimal solution to the mean-variance model, which is widely used for the portfolio optimization problem; in particular, we analyzed its potential investment risk and the concentrated investment level using self-averaging and replica analysis. We used the example of the rock-paper-scissors game with two subjects as a context for the optimization of stochastic phenomena. We noted that the minimal expected investment risk (from our discussion of an annealed disorder system) is not always in agreement with the expected minimal investment risk (from our discussion of a quenched disorder system). We discussed whether the optimal investment strategy which was derived using the analytical procedure that is widely used in operations research and the maximization of the expected utility based on an annealed disorder system are valid for use with actual return rates on assets. From the relationship in Eq (7), based on the more general formulation, we determined that the minimal expected investment risk obtained by the operations research approach was not smaller than the expected minimal investment risk. However, it does not provide useful information for an investment strategy since it underestimates the expected minimal investment risk. The main reasons for this are as follows. (1) At the start of an investment, there is no a priori knowledge of the future return rates on the assets. (2) The computational complexity required to assess the inverse of the optimal solution matrix increases with the cube of the number of investment outlets. (3) In order to precisely assess the potential investment risk, it is necessary to average the minimal investment risk with the actual return rate. In order to solve these problems, we used probabilistic reasoning to reformulate the portfolio optimization problem; we also used the Chernoff inequality and replica analysis to determine a tighter upper bound for the cumulative distribution of the investment risk. From an analytical result for the rate function that was derived from replica analysis, we clarified the self-averaging property of the investment risk. Thus, we determined that the minimal investment risk for the case in which complete information on the return rates is known a priori is in agreement with the minimal investment risk for the case in which the return rate matrix is averaged. We are thus able to evaluate the potential investment risk in an actual investment system. From this, we have solved the first and third problems that we listed above. Furthermore, by using replica analysis, we estimated two indicators for the optimal portfolio: the investment risk and the concentrated investment level; this was done without resolving the optimal portfolio directly, and this resolved the second problem. We found that the concentrated investment level obtained by our proposed approach was consistent with the intuitively obvious choice for an optimal investment strategy; we considered cases in which the scenario ratio approached 1 and in which the ratio was sufficiently large. We compared the results of our proposed method, the results obtained by the operations research approach, and the results obtained from a numerical simulation. The results of our method were in agreement with the results of the numerical simulation, but they did not coincide with the results of the operations research approach. As discussed above, although our findings are based on the mean-variance model with only a budget constraint and a return rate which is independently and identically distributed with a standard normal distribution, the relationship in Eq (7) and our findings imply that the approach based on maximizing the expected utility is not able to determine the most desirable strategy for actual investments.

In our future work, although for simplicity we considered only a budget constraint in this paper, in order to make our method more realistic, we wish to determine the optimal solutions under other constraints, such as limits on expected gross earnings, short-selling restrictions, and upper and lower limits for each asset. In particular, we wish to consider whether these problems can be resolved by using other analytical approaches of statistical mechanical informatics, such as the belief propagation method, random matrix integrals, or the Markov Chain Monte Carlo method. It is also necessary to confirm the self-averaging property of the risk function for cases other than the mean-variance model, such as for the mean-absolute deviation model or the expected shortfall model (for these models, typical behaviours of investment risk were evaluated by Ciliberti and Mézard). In addition, in order to clarify the mathematical structure of this optimization problem, we assumed that the return rates on assets were independently and identically distributed with a standard normal distribution, however, in an actual investment market, the return rate is not always independently and identically distributed. We would thus like to quantify the effects of this correlation on the indicators. Thus, although several models used in operations research have been proposed for assessing investment systems, in many cases, only the expected utility has been maximized; that is, only the annealed disorder system has been analyzed. The portfolio optimization problem is an undeveloped field, and many issues have not yet been considered.

## Appendix

## 1. Properties of the rate function

We introduce the properties of the rate function *R*(*η*); these properties support the discussion in above section. In this appendix, for convenience, we define the function

$$\begin{array}{ccc} \phi (u) & = & \log E[e^{uY}]. \end{array}$$ (57)

Moreover, we will discuss only *Pr*[*η* ≤ *Y*] ≤ *e* ^−*R*(*η*)^ and *R*(*η*) = max_*u* > 0_{*uη* − *ϕ*(*u*)}, but we note that *Pr*[*η* ≥ *Y*] ≤ *e* ^−*R*(*η*)^ and *R*(*η*) = max_*u* < 0_{*uη* − *ϕ*(*u*)} can be verified in a similar way.

### *R*(*η*) ≥ 0 is held

For any *η* ∈ **R**, *R*(*η*) ≥ 0 holds. Also, for any *u* > 0, *R*(*η*) = max_*u* > 0_{*uη* − *ϕ*(*u*)} ≥ *uη* − *ϕ*(*u*) = *R*(*η*, *u*) in the limit as *u* goes to +0, that is, we obtain lim_*u* → +0_ *R*(*η*, *u*) = 0 for *R*(*η*) ≥ 0. In addition, if *R*(*η*) < 0, since *Pr*[*η* ≤ *Y*] ≤ 1 < *e* ^−*R*(*η*)^, *R*(*η*) ≥ 0 implies an intuitive upper bound for the cumulative distribution.

### When *E*[*Y*] ≥ *η*, *R*(*η*) = 0

When *η* is less than or equal to *E*[*Y*], the expectation of *Y*, that is, *E*[*Y*] ≥ *η*, then *R*(*η*) = 0. Since *e* ^*uY*^ is a convex function of *Y*, for any *u* > 0, *ϕ*(*u*) ≥ log*e* ^*uE*[*Y*]^ = *uE*[*Y*], and we obtain 0 ≥ *uE*[*Y*] − *ϕ*(*u*). If *η* = *E*[*Y*], then we obtain *R*(*E*[*Y*]) = 0 from *R*(*E*[*Y*], *u*) = *uE*[*Y*] − *ϕ*(*u*) ≤ 0. In addition, if *E*[*Y*] ≥ *η*, then 0 ≥ *uE*[*Y*] − *ϕ*(*u*) ≥ *uη* − *ϕ*(*u*) = *R*(*η*, *u*), and we obtain *R*(*η*) = 0. That is, this property may intuitively imply *E*[*Y*] = sup{*η*∣*R*(*η*) = 0}.

### *R*(*η*) is a convex function

*R*(*η*), which is derived from the Legendre transformation of a convex function, is a convex function of *η*. Thus, for any ∀*λ* ∈ [0,1), *R*(*η*) and *R*(*ξ*),

$$\begin{array}{ccc} \lambda R(\eta )+(1-\lambda )R(\xi ) & \geq & \lambda R(\eta ,u)+(1-\lambda )R(\xi ,u)\\ & = & u(\lambda \eta +(1-\lambda )\xi )-\phi (u)\\ & = & R(\lambda \eta +(1-\lambda )\xi ,u), \end{array}$$ (58)

where *u* is nonnegative. Thus, the *λR*(*η*) + (1 − *λ*)*R*(*ξ*) ≥ *R*(*λη* + (1 − *λ*)*ξ*) is obtained by maximizing both sides of Eq (58) for *u* > 0.

## 2. Calculation of replica analysis

In this appendix, we analytically evaluate *E*[*Z* ^*n*^(*β*, *X*)] using replica analysis; however, in general, it is difficult to estimate *E*[*Z* ^*n*^(*β*, *X*)] for any *n* ∈ **R** [10]. Direct evaluation of *E*[*Z* ^*n*^], the *n*-th moment of a nonnegative random variable *Z* ≥ 0, at any *n* ∈ **R**, is not possible unless the random variable follows a log-normal distribution [18, 19]. In particular, it is not easy to assess the partition function *Z*(*β*, *X*), defined in integral form in Eq (16), with a fixed return rate matrix *X*. If we could calculate this directly, we could easily solve Eqs (24) and (25) without needing to refer to the Helmholtz free energy; however, it is difficult to directly evaluate a partition function with a fixed return matrix in this model. We can solve *E*[*Z* ^*n*^(*β*, *X*)] with the replica number *n* ∈ **N** because it is comparatively easy to calculate *E*[*Z* ^*n*^(*β*, *X*)] for any replica number *n* ∈ **N**, and this can be used to estimate *E*[*Z* ^*n*^(*β*, *X*)] at any replica number *n* ∈ **R**. Intuitively, for instance, it is possible to expand (*a* + *b*)^2^ = *a* ^2^+2*ab* + *b* ^2^ and (*a* + *b*)^3^ = *a* ^3^+3*a* ^2^ *b*+3*ab* ^2^+*b* ^3^ with finite terms, although it is not possible to obtain a finite expansion of (*a* + *b*)^2.5^. Nevertheless, it is trivial that this expansion will be between the square and the cube of (*a* + *b*), that is, (*a* + *b*)^2^ < (*a* + *b*)^2.5^ < (*a* + *b*)^3^. Thus, as a first step, we can estimate *E*[*Z* ^*n*^(*β*, *X*)] at replica number *n* ∈ **N**, and then use this to estimate *E*[*Z* ^*n*^(*β*, *X*)] at replica number *n* ∈ **R**. This approach is called replica analysis.

We can evaluate *E*[*Z* ^*n*^(*β*, *X*)] at *n* ∈ **N**, as follows:

$$\begin{array}{ccc} E[Z^{n}\left(\beta ,X\right)] & = & E[{\left(\int_{-\infty }^{\infty }dwP_{0}\left(w\right)e^{-\beta 𝓗(w|X)}\right)}^{n}]\\ & = & \int_{-\infty }^{\infty }\prod_{a=1}^{n}dw_{a}P_{0}\left(w_{a}\right)\\ & & E[\exp(-\frac{\beta }{2}\sum_{\mu =1}^{p}\sum_{a=1}^{n}{\left(\frac{1}{\sqrt{N}}\sum_{i=1}^{N}x_{i\mu }w_{ia}\right)}^{2})], \end{array}$$ (59)

where **w**_*a*_ = (*w*_1*a*_, ⋯, *w*_*Na*_)^T^ ∈ **R** ^*N*^, (*a* = 1, 2, ⋯, *n*). Moreover, since *P*_0_(**w**_*a*_) is the coefficient used to average the return rate matrix *X*, it can be separated.

We now introduce the Dirac delta function *δ*(*x*) in order to use it to average the *x*_*kμ*_. The Dirac delta function *δ*(*x*) is one of the most widely used generalized functions, defined for any *f*(*x*) as

$$\begin{array}{ccc} f(w) & = & \int_{-\infty }^{\infty }dvf\left(v\right)\delta \left(v-w\right). \end{array}$$ (60)

This function returns the function *f*(*v*) when the argument of *δ*(*v* − *w*) on the right-hand side, *v* − *w*, is 0, that is *f*(*w*). Thus, if a constant function is used in Eq (60), for example, *f*(*x*) = 1, then

$$\begin{array}{ccc} \int_{-\infty }^{\infty }dz\delta \left(z\right) & = & 1. \end{array}$$ (61)

In addition, the Fourier transform of the Dirac delta function,

$$\begin{array}{ccc} \delta (z) & = & \frac{1}{2\pi }\int_{-\infty }^{\infty }{due}^{iuz}, \end{array}$$ (62)

can be obtained if the imaginary unit $i=\sqrt{-1}$ is employed. Thus, the integrand in Eq (59) can be written as

$$\begin{array}{ccc} & & \exp(-\frac{\beta }{2}{\left(\frac{1}{\sqrt{N}}\sum_{i=1}^{N}x_{i\mu }w_{ia}\right)}^{2})\\ & = & \frac{1}{2\pi }\int_{-\infty }^{\infty }{dv}_{\mu a}du_{\mu a}\exp(-\frac{\beta v_{\mu a}^{2}}{2}+iu_{\mu a}(v_{\mu a}-\frac{1}{\sqrt{N}}\sum_{i=1}^{N}x_{i\mu }w_{ia})). \end{array}$$ (63)

Substituting this into Eq (59), we obtain

$$\begin{array}{ccc} E[Z^{n}\left(\beta ,X\right)] & = & \frac{1}{{\left(2\pi \right)}^{pn}}\int_{-\infty }^{\infty }\prod_{a=1}^{n}dw_{a}P_{0}\left(w_{a}\right)du_{a}dv_{a}\\ & & \exp(i\sum_{\mu =1}^{p}\sum_{a=1}^{n}u_{\mu a}v_{\mu a}-\frac{\beta }{2}\sum_{\mu =1}^{p}\sum_{a=1}^{n}v_{\mu a}^{2})\\ & & E_{X}[\exp(-\frac{i}{\sqrt{N}}\sum_{\mu =1}^{p}\sum_{a=1}^{n}\sum_{i=1}^{N}u_{\mu a}x_{i\mu }w_{ia})], \end{array}$$ (64)

where **u**_*a*_ = (*u*_1*a*_, ⋯, *u*_*pa*_)^T^ ∈ **R** ^*p*^ and **v**_*a*_ = (*v*_1*a*_, ⋯, *v*_*pa*_)^T^ ∈ **R** ^*p*^, (*a* = 1, 2, ⋯, *n*). Since the return rate *x*_*iμ*_ is independently and identically distributed with a standard normal distribution, the expectation of *x*_*iμ*_ is

$$\begin{array}{ccc} E[\exp(-\frac{ix_{i\mu }}{\sqrt{N}}\sum_{a=1}^{n}u_{\mu a}w_{ia})] & = & \exp(-\frac{1}{2N}{\left(\sum_{a=1}^{n}u_{\mu a}w_{ia}\right)}^{2}) \end{array}$$ (65)

where $\int_{-\infty }^{\infty }\frac{dx}{\sqrt{2\pi \sigma^{2}}}e^{-\frac{{\left(x-m\right)}^{2}}{2\sigma^{2}}+ix\theta }=e^{im\theta -\frac{\sigma^{2}\theta^{2}}{2}}$. Thus we obtain

$$\begin{array}{ccc} E[Z^{n}\left(\beta ,X\right)] & = & \frac{1}{{\left(2\pi \right)}^{pn}}\int_{-\infty }^{\infty }\prod_{a=1}^{n}dw_{a}P_{0}\left(w_{a}\right)du_{a}dv_{a}\\ & & \exp(i\sum_{\mu =1}^{p}\sum_{a=1}^{n}u_{\mu a}v_{\mu a}-\frac{\beta }{2}\sum_{\mu =1}^{p}\sum_{a=1}^{n}v_{\mu a}^{2}\\ & & -\frac{1}{2N}\sum_{\mu =1}^{p}\sum_{i=1}^{N}{\left(\sum_{a=1}^{n}u_{\mu a}w_{ia}\right)}^{2}). \end{array}$$ (66)

We then substitute

$$\begin{array}{ccc} q_{wab} & = & \frac{1}{N}\sum_{i=1}^{N}w_{ia}w_{ib} \end{array}$$ (67)

and obtain

$$\begin{array}{ccc} -\frac{1}{2N}\sum_{\mu =1}^{p}\sum_{i=1}^{N}{\left(\sum_{a=1}^{n}u_{\mu a}w_{ia}\right)}^{2} & = & -\frac{1}{2}\sum_{\mu =1}^{p}\sum_{a=1}^{n}\sum_{b=1}^{n}u_{\mu a}u_{\mu b}q_{wab}. \end{array}$$ (68)

From this technique, we obtain

$$\begin{array}{ccc} E[Z^{n}\left(\beta ,X\right)] & = & \underset{Q_{w},{\tilde{Q}}_{w}}{\mathrm{Extr}}\{\frac{1}{{\left(2\pi \right)}^{pn}}\int_{-\infty }^{\infty }\prod_{a=1}^{n}dw_{a}P_{0}\left(w_{a}\right)du_{a}dv_{a}\\ & & \exp(i\sum_{\mu =1}^{p}\sum_{a=1}^{n}u_{\mu a}v_{\mu a}-\frac{\beta }{2}\sum_{\mu =1}^{p}\sum_{a=1}^{n}v_{\mu a}^{2}-\frac{1}{2}\sum_{\mu =1}^{p}\sum_{a,b}u_{\mu a}u_{\mu b}q_{wab}\\ & & -\frac{1}{2}\sum_{a,b}{\tilde{q}}_{wab}(\sum_{i=1}^{N}w_{ia}w_{ib}-Nq_{wab}))\}, \end{array}$$ (69)

where ∑_*a*, *b*_ means $\sum_{a=1}^{n}\sum_{b=1}^{n}$ and Extr_*A*_ *f*(*A*) are the extrema of *f*(*A*) with respect to *A*. In order to satisfy the constraint in Eq (67), we use the auxiliary variable ${\tilde{q}}_{wab}$. Moreover *Q*_*w*_ = {*q*_*wab*_} ∈ 𝓜_*n* × *n*_ and ${\tilde{Q}}_{w}=\left\{{\tilde{q}}_{wab}\right\}\in {𝓜}_{n\times n}$ are the order parameter matrices.

We can separate the integral of *u*_*μa*_, *v*_*μa*_ from the integral of *w*_*ka*_. We evaluate the integral of *u*_*μa*_, *v*_*μa*_,

$$\begin{array}{ccc} & & \frac{1}{{\left(2\pi \right)}^{pn}}\int_{-\infty }^{\infty }\prod_{\mu =1}^{p}\prod_{a=1}^{n}du_{\mu a}dv_{\mu a}\\ & & \exp(\sum_{\mu =1}^{p}(i\sum_{a=1}^{n}u_{\mu a}v_{\mu a}-\frac{\beta }{2}\sum_{a=1}^{n}v_{\mu a}^{2}-\frac{1}{2}\sum_{a,b}u_{\mu a}u_{\mu b}q_{wab}))\\ & & \;={\left\{\frac{1}{{\left(2\pi \right)}^{n}}\int_{-\infty }^{\infty }dudve^{iu^{T}v-\frac{\beta }{2}v^{T}v-\frac{1}{2}u^{T}Q_{w}u}\right\}}^{p}\\ & & \;=\exp[-\frac{p}{2}\log\det|I+\beta Q_{w}|], \end{array}$$ (70)

where *I* ∈ 𝓜_*n* × *n*_ is the identity matrix. Because this is independent of the scenario index *μ*, we can estimate the integral using two novel vectors, **u** = (*u*_1_, ⋯, *u*_*n*_)^T^ ∈ **R** ^*n*^, and **v** = (*v*_1_, ⋯, *v*_*n*_)^T^ ∈ **R** ^*n*^. On the other hand, we can calculate the integral of *w*_*ka*_,

$$\begin{array}{ccc} & & \int_{-\infty }^{\infty }\prod_{k=1}^{N}\prod_{a=1}^{n}dw_{ka}P_{0}\left(w_{a}\right)\exp(-\frac{1}{2}\sum_{k=1}^{N}\sum_{a,b}{\tilde{q}}_{wab}w_{ka}w_{kb}+\frac{N}{2}\text{Tr}Q_{w}{\tilde{Q}}_{w})\\ & & \;=\underset{k}{\mathrm{Extr}}\frac{1}{{\left(2\pi \right)}^{\frac{Nn}{2}}}\int_{-\infty }^{\infty }\prod_{k=1}^{N}\prod_{a=1}^{n}dw_{ka}\\ & & \;\exp(\sum_{a=1}^{n}k_{a}(\sum_{k=1}^{N}w_{ka}-N)-\frac{1}{2}\sum_{k=1}^{N}\sum_{a,b}{\tilde{q}}_{wab}w_{ka}w_{kb}+\frac{N}{2}\text{Tr}Q_{w}{\tilde{Q}}_{w})\\ & & \;=\underset{k}{\mathrm{Extr}}\exp[-Nk^{T}e+\frac{N}{2}\text{Tr}Q_{w}{\tilde{Q}}_{w}]{\left\{\frac{1}{{\left(2\pi \right)}^{\frac{n}{2}}}\int_{-\infty }^{\infty }dwe^{-\frac{1}{2}w^{T}\tilde{Q}w+k^{T}w}\right\}}^{N}\\ & & \;=\underset{k}{\mathrm{Extr}}\exp[-Nk^{T}e+\frac{N}{2}\text{Tr}Q_{w}{\tilde{Q}}_{w}-\frac{N}{2}\log\det|{\tilde{Q}}_{w}|+\frac{N}{2}k^{T}{\tilde{Q}}_{w}^{-1}k], \end{array}$$ (71)

where **k** = (*k*_1_, ⋯, *k*_*n*_)^T^ ∈ **R** ^*n*^,**e** = (1, ⋯, 1)^T^ ∈ **R** ^*n*^, **w** is the prior probability of the portfolio, *P*_0_(**w**_*a*_) is replaced by ${\text{Extr}}_{k_{a}}\text{exp}\left(k_{a}\left(\sum_{k=1}^{N}w_{ka}-N\right)-\frac{N}{2}\text{log}2\pi \right)$, and because this is not dependent on the asset index *k*, we can solve the integral using a novel vector **w** = (*w*_1_, ⋯, *w*_*n*_)^T^ ∈ **R** ^*n*^.

We summarize this and rewrite the limit of $\frac{1}{N}\text{log}E[Z^{n}(\beta ,X)]$ for the number of investment outlets *N* as Φ(*n*):

$$\begin{array}{ccc} \Phi (n) & = & \lim_{N\to \infty }\frac{1}{N}\log E\left[Z^{n}\left(\beta ,X\right)\right]\\ & = & \underset{k,Q_{w},{\tilde{Q}}_{w}}{\mathrm{Extr}}\{-\frac{\alpha }{2}\log\det|I+\beta Q_{w}|+\frac{1}{2}\text{Tr}Q_{w}{\tilde{Q}}_{w}-\frac{1}{2}\log\det|{\tilde{Q}}_{w}|\\ & & -k^{T}e+\frac{1}{2}k^{T}{\tilde{Q}}_{w}^{-1}k\}. \end{array}$$ (72)

Although a sufficiently large number of investment outlets *N* is required to guarantee that evaluating by using the order parameters $k_{a},q_{wab},{\tilde{q}}_{wab}$ as defined in Eqs (69) and (71) is consistent with the constraints of Eqs (67) and (2) in the replica analysis, our target indicator *ɛ* represents the minimal investment risk per asset; that is, since this is independent of the system size *N*, there will not be problems in the limit as *N* approaches infinity. It is preferable to normalize the investment risk per asset with respect to different sizes of investment markets, and this allows the comparison of potential investment risks.

We note two important points. First, in above subsection, we already mentioned that *ɛ*(*X*) = *E*[*ɛ*(*X*)], since the investment risk is self-averaging. From the above discussion, we have verified that

$$\begin{array}{ccc} E[\varepsilon (X)] & = & -\lim_{\beta \to \infty }\frac{\partial }{\partial \beta }\{\lim_{N\to \infty }\frac{1}{N}E\left[\log Z\left(\beta ,X\right)\right]\}\\ & = & -\lim_{\beta \to \infty }\frac{\partial }{\partial \beta }\{\lim_{n\to 0}\frac{\partial \Phi (n)}{\partial n}\}\\ & = & -\lim_{\beta \to \infty }\frac{\partial }{\partial \beta }\{-\frac{\alpha }{2}\log\frac{\alpha }{\alpha -1}-\frac{\beta (\alpha -1)}{2}+\frac{1}{2}-\frac{1}{2}\log\beta \left(\alpha -1\right)\}\\ & = & \frac{\alpha -1}{2}, \end{array}$$ (73)

where we assume that the replica number *n* is a continuous number, and we use the replica trick $E[\text{log}Z]={\text{lim}}_{n\to 0}\frac{\partial }{\partial n}\text{log}E[Z^{n}]$[10, 18, 19]. This result is consistent with that of Eq (51). Second, from the definition in Eq (67), since *q*_*waa*_ is consistent with the concentrated investment level *q*_*w*_ in Eq (5), $q_{w}=q_{waa}=\frac{1}{\beta (\alpha -1)}+\frac{\alpha }{\alpha -1}$. However, since this is an optimal solution with a sufficiently large *β*,

$$\begin{array}{ccc} q_{w} & = & \frac{\alpha }{\alpha -1}. \end{array}$$ (74)

Although we used replica analysis to analyze the minimal investment risk, we can also obtain the concentrated investment level of the optimal portfolio. Fortunately, in the limit of very large *N*, *q*_*w*_ is finite; thus there is an advantage of using Eq (2) as the budget constraint.

## 3. Random matrix approach for minimal investment risk and concentrated investment level

We show here that it is also possible to evaluate the two indicators, *ɛ* and *q*_*w*_, by using an asymptotic eigenvalue distribution of a random matrix [9]. As in the above discussion, we will consider only the case *α* = *p*/*N* > 1 in order to uniquely determine the optimal solution of Eq (1). Using the optimal solution defined in Eq (3), from Eqs (13) and (14), the minimal investment risk per asset *ɛ* and the concentrated investment level *q*_*w*_ are replaced, as follows:

$$\begin{array}{ccc} \varepsilon & = & \frac{1}{2(\frac{1}{N}e^{T}J^{-1}e)}, \end{array}$$ (75)

$$\begin{array}{ccc} q_{w} & = & \frac{\left(\frac{1}{N}e^{T}J^{-2}e\right)}{{\left(\frac{1}{N}e^{T}J^{-1}e\right)}^{2}}. \end{array}$$ (76)

If *N* is sufficiently large, we have

$$\begin{array}{ccc} \varepsilon & = & \frac{1}{2g(1)}, \end{array}$$ (77)

$$\begin{array}{ccc} q_{w} & = & \frac{g(2)}{{\left(g(1)\right)}^{2}}, \end{array}$$ (78)

where $g(s)={\text{lim}}_{N\to \infty }\frac{1}{N}{\text{e}}^{\text{T}}J^{-s}\text{e}$. If we could analyze *g*(*s*), then *ɛ* and *q*_*w*_ could be precisely determined. It turns out that it is easy to assess *g*(*s*) by using a random matrix ensemble.

For this ensemble of random matrices, we require the following two properties: (1) when the random matrix $X=\left\{\frac{x_{k\mu }}{\sqrt{N}}\right\}\in {𝓜}_{N\times p}$ is decomposed as *X* = *UDV*, where *U* ∈ 𝓜_*N* × *N*_ and *V* ∈ 𝓜_*p* × *p*_ are orthogonal matrices and *D* ∈ 𝓜_*N* × *p*_ is a diagonal rectangular matrix, then *U* and *V* are independently distributed with a Haar measure; (2) when *N* is sufficiently large, the distribution of the eigenvalues of the variance-covariance matrix *J* = *XX* ^T^, for any return rate matrix *X*, is asymptotically close to $\rho (\lambda )={\text{lim}}_{N\to \infty }\sum_{k=1}^{N}\delta (\lambda -\lambda_{k})$, where *λ*_*k*_ is the *k*th diagonal of *DD* ^T^ = diag{*λ*_1_, *λ*_2_, ⋯, *λ*_*N*_} ∈ 𝓜_*N* × *N*_; if *N* and *p* simultaneously approach infinity, then it is required that *α* = *p*/*N* ∼ *O*(1). If these two properties are satisfied, then

$$\begin{array}{ccc} g(s) & = & \int_{0}^{\infty }d\lambda \rho \left(\lambda \right)\lambda^{-s}. \end{array}$$ (79)

Moreover, if the return rates are independently and identically distributed with a standard normal distribution, then the random matrix *X* satisfies the requirements for the random matrix ensemble described above [9, 20].

Next, we consider the asymptotic eigenvalue distribution. If the return rate *x*_*kμ*_ is independently and identically distributed, its mean and variance are respectively 0 and 1, and the higher-order moments are finite, that is, ∣*E*[(*x*_*kμ*_)^*s*^]∣ < ∞, (*s* = 3, 4, ⋯), then the distribution of the eigenvalues of the variance-covariance matrix *J* = *XX* ^T^ of the return rate matrix $X=\left\{\frac{x_{k\mu }}{\sqrt{N}}\right\}\in {𝓜}_{N\times p}$ is asymptotically close to

$$\begin{array}{ccc} \rho (\lambda ) & = & {\left[1-\alpha \right]}^{+}\delta \left(\lambda \right)+\frac{\sqrt{{\left[\lambda -\lambda_{-}\right]}^{+}{\left[\lambda_{+}-\lambda \right]}^{+}}}{2\pi \lambda }, \end{array}$$ (80)

where *δ*(*u*) is the Dirac delta function, [*u*]^+^ = max(0, *u*), and $\lambda_{\pm }=1+\alpha \pm 2\sqrt{\alpha }$ [21–23]. This eigenvalue distribution *ρ*(*λ*) is called the Marčenko-Pastur law, and this distribution can be regarded as the limit distribution for the eigenvalues, similar to the limit distribution (normal distribution) guaranteed by the central limit theorem.

The eigenvalues in this distribution can be easily calculated:

$$\begin{array}{ccc} g(1) & = & \frac{\lambda_{+}+\lambda_{-}}{4\sqrt{\lambda_{+}\lambda_{-}}}-\frac{1}{2}\\ & = & \frac{1}{\alpha -1} \end{array}$$ (81)

$$\begin{array}{ccc} g(2) & = & \frac{\sqrt{\lambda_{+}\lambda_{-}}}{4}{\left(\frac{\frac{1}{\lambda_{-}}-\frac{1}{\lambda_{+}}}{2}\right)}^{2}\\ & = & \frac{\alpha }{{\left(\alpha -1\right)}^{3}}, \end{array}$$ (82)

where

$$\begin{array}{ccc} \int \frac{dx}{\sqrt{ax^{2}+bx+c}} & = & -\frac{1}{\sqrt{\left|a\right|}}\sin^{-1}\frac{2ax+b}{\sqrt{b^{2}-4ac}},\;\left(a<0\right) \end{array}$$ (83)

$$\begin{array}{ccc} \int \frac{dx}{x\sqrt{ax^{2}+bx+c}} & = & \frac{1}{\sqrt{\left|c\right|}}\sin^{-1}\frac{2c+bx}{x\sqrt{b^{2}-4ac}},\;\left(c<0\right). \end{array}$$ (84)

Thus,

$$\begin{array}{ccc} \varepsilon & = & \frac{\alpha -1}{2} \end{array}$$ (85)

$$\begin{array}{ccc} q_{w} & = & \frac{\alpha }{\alpha -1}. \end{array}$$ (86)

This result is consistent with the result we obtained by replica analysis and numerical simulation.

## Supporting Information

S1 Data Figure 1 from S1 Data. (DOCX)
