# Supplementary material for: Self-Averaging Property of Minimal Investment Risk of Mean-Variance Model
Source: PLoS One. 2015 Jul 30;10(7):e0133846. doi: 10.1371/journal.pone.0133846 (PMC4520490; doi:10.1371/journal.pone.0133846)
Supplement: S1 Data — (DOCX) [file pone.0133846.s001.docx]

8.000000 1.142093 0.007884 3.501245 0.061181

7.800000 1.146032 0.007922 3.401248 0.060530

7.600000 1.150314 0.007986 3.303299 0.061185

7.400000 1.155141 0.007783 3.202627 0.061273

7.200000 1.160371 0.007697 3.101263 0.060063

7.000000 1.165626 0.007429 3.001954 0.059029

6.800000 1.171330 0.007949 2.902117 0.056068

6.600000 1.177562 0.008402 2.801452 0.053505

6.400000 1.184507 0.008736 2.699392 0.052468

6.200000 1.191844 0.009476 2.600132 0.051825

6.000000 1.199088 0.009590 2.501663 0.051896

5.800000 1.207108 0.009518 2.402242 0.050889

5.600000 1.216141 0.010037 2.302186 0.048735

5.400000 1.226240 0.010213 2.201028 0.049577

5.200000 1.237286 0.010517 2.102406 0.049593

5.000000 1.248933 0.010930 2.004316 0.048256

4.800000 1.261962 0.011837 1.902187 0.047602

4.600000 1.277501 0.013045 1.801361 0.045850

4.400000 1.294279 0.013716 1.700045 0.043943

4.200000 1.312273 0.014653 1.600460 0.043831

4.000000 1.334063 0.016737 1.499790 0.042192

3.800000 1.357675 0.019076 1.400575 0.041183

3.600000 1.384172 0.019908 1.300711 0.038254

3.400000 1.417002 0.020670 1.200551 0.039387

3.200000 1.456707 0.021404 1.099126 0.037904

3.000000 1.501659 0.023532 0.999067 0.035555

2.800000 1.556106 0.029335 0.899602 0.033746

2.600000 1.624913 0.032430 0.800562 0.031092

2.400000 1.715437 0.039869 0.700677 0.027335

2.200000 1.838688 0.049365 0.599361 0.024907

2.000000 2.004547 0.066771 0.500435 0.023365

1.800000 2.250386 0.080386 0.400756 0.021452

1.600000 2.659461 0.115817 0.301992 0.018322

1.400000 3.489665 0.220842 0.202549 0.014622

1.200000 6.014912 0.611293 0.102071 0.010068
